# The contribution of genetic variants of SLC2A1 gene in T2DM and T2DM-nephropathy: association study and meta-analysis

**DOI:** 10.1080/0886022X.2018.1496931

**Published:** 2018-10-24

**Authors:** I. Stefanidis, M. Tziastoudi, E. E. Tsironi, E. Dardiotis, S. V. Tachmitzi, A. Fotiadou, G. Pissas, K. Kytoudis, M. Sounidaki, G. Ampatzis, P. R. Mertens, V. Liakopoulos, T. Eleftheriadis, G. M Hadjigeorgiou, M. Santos, E. Zintzaras

**Affiliations:** a Department of Nephrology, University of Thessaly School of Medicine, Larissa, Greece;; b Department of Biomathematics, University of Thessaly School of Medicine, Larissa, Greece;; c Department of Ophthalmology, University of Thessaly School of Medicine, Larissa, Greece;; d Department of Neurology, University of Thessaly School of Medicine, Larissa, Greece;; e Department of Nephrology, Hypertension, Diabetes and Endocrinology, School of Medicine, University of Magdeburg, Magdeburg, Germany;; f Departament de Genètica i de Microbiologia, Universitat Autònoma de Barcelona, Bellaterra, Spain;; g The Institute for Clinical Research and Health Policy Studies, Tufts Medical Center, Tufts University School of Medicine, Boston, MA, USA

**Keywords:** Diabetes mellitus, diabetic nephropathy, glucose transporter 1 (GLUT1), genetic variants of SLC2A1

## Abstract

An association study was conducted to investigate the relation between 14 variants of glucose transporter 1 gene (*SLC2A1*) and the risk of type 2 diabetes (T2DM) leading to nephropathy. We also performed a meta-analysis of 11 studies investigating association between diabetic nephropathy (DN) and *SLC2A1* variants. The cohort included 197 cases (T2DM with nephropathy), 155 diseased controls (T2DM without nephropathy) and 246 healthy controls. The association of variants with disease progression was tested using generalized odds ratio (OR_G_). The risk of type 2 diabetes leading to nephropathy was estimated by the OR of additive and co-dominant models. The mode of inheritance was assessed using the degree of dominance index (h-index). We synthesized results of 11 studies examining association between 5 *SLC2A1* variants and DN. OR_G_ was used to assess the association between variants and DN using random effects models. Significant results were derived for co-dominant model of rs12407920 [OR = 2.01 (1.17–3.45)], rs841847 [OR = 1.73 (1.17–2.56)] and rs841853 [OR = 1.74 (1.18–2.55)] and for additive model of rs3729548 [OR = 0.52 (0.29–0.90)]. The mode of inheritance for rs12407920, rs841847 and rs841853 was ‘dominance of each minor allele’ and for rs3729548 ‘non-dominance’. Frequency of one haplotype (C-G-G-A-T-C-C-T-G-T-C-C-A-G) differed significantly between cases and healthy controls [*p* = .014]. Regarding meta-analysis, rs841853 contributed to an increased risk of DN [(OR_G_ = 1.43 (1.09–1.88); OR_G_ = 1.58 (1.01–2.48)] between diseased controls versus cases and healthy controls versus cases, respectively. Further studies confirm the association of rs12407920, rs841847, rs841853, as well as rs3729548 and the risk of T2DM leading to nephropathy.

## Introduction

Diabetic nephropathy is a major microvascular complication of diabetes mellitus [1]. It is the most frequent primary cause of end-stage renal disease and is characterized by a progressive clinical course, ultimately leading to death [2]. The main risk factor for developing diabetic nephropathy or any microvascular complication in diabetes is the poor glycemic control; though patients with good glycemic control may develop nephropathy [1]. This fact and a proven significant familial clustering of diabetic nephropathy [3–5] clearly implicate that specific genetically defined predisposition is involved in the pathogenesis of nephropathy in diabetes. However, the genes conferring susceptibility have not been identified yet [6–11].

The glucose transporter 1 (GLUT1), also called *SLC2A1*, i.e., the member 1 of the solute carriers family 2, is a most important representative of the facilitative glucose transporters. Its expression in the glomerular mesangial cell membrane is rate-limiting for intracellular glucose flux and utilization [12,13]. In mesangial cells, elevated levels of intracellular glucose, e.g., resulting from diabetes mellitus, activates cellular pathways involved in cellular growth and in the accumulation of extracellular matrix [14–16]. Exactly, these alterations are central in the pathogenesis of diabetic nephropathy. Mesangial cells over-expressing GLUT1 after gene transduction *in vitro* acquire a diabetic phenotype with accumulation of extracellular matrix even in the absence of enhanced glucose levels in the medium [13,17]. Furthermore, transgenic mice over-expressing GLUT1 on kidney glomerular cells develop diabetic nephropathy, despite normoglycemia [18]. Thus, it appears that the availability of GLUT1 transporters, rather than extracellular glucose concentrations per se, regulates mesangial cell glucose metabolic flux. In support of this contention, reduction of GLUT1 expression in mesangial cells [19] and in transgenic diabetic (db) mice [12,20] protects against diabetic complications, despite high glucose concentration. From these data, it becomes clear that GLUT1 on the cell membrane of glomerular cells may possess a central regulatory role for the development of diabetic nephropathy.

In the present candidate-gene study, we tested the hypothesis of association between 14 variants of *SLC2A1* (rs12407920 C/T, rs2297976 G/T, rs710221 G/A, rs2086856 A/G, rs12130264 C/T, rs841847 C/T, rs841853 C/A, rs3729548 C/T, rs841855 G/A, rs3768029 C/T, rs12071418 C/G, rs3820549 C/G, rs3820546 G/A, rs11537641 G/A) and the progression of type 2 diabetes (i.e., from healthy status to diabetes without nephropathy and then, to diabetes leading to nephropathy). Thereafter, we tested the association between the *SLC2A1* variants and the risk of diabetes leading to nephropathy. The former hypothesis was tested by the generalized linear odds ratio (OR_G_) [21,22]. The latter hypothesis was tested using the OR_G_ as a genetic model-free approach and also by means of the additive and co-dominant inheritance models [21–23]. In addition, the mode of inheritance was estimated based on the degree of dominance index (h-index) [24,25]. Finally, an analysis of haplotypes was conducted.

To further investigate the contribution of *SLC2A1* polymorphisms in the development and progression of DN, we performed a meta-analysis of all variants across *SLC2A1* that had been examined in genetic association studies up-to-date. The variants included in meta-analysis were rs841853, rs1385129, rs841847, rs841848 and rs710218, out of which variants rs841853 and rs841847 were also genotyped in present case-control study.

## Methods

### Association study

#### Subjects

Ethics Committee of the University Hospital of Larissa, University of Thessaly, School of Medicine approved the study protocol. The study was conducted in the University Hospital of Larissa and all participants signed an informed consent before enrolment. All participants were recruited from patients attending the outpatient wards of Nephrology, Internal Medicine and Ophthalmology at the University Hospital of Larissa between 2009 and 2011. They were all Caucasians of Greek origin and during the study, they resided in the same region in central Greece (Thessaly).

The study cohort consisted of cases with type 2 diabetes and nephropathy, diseased controls (type 2 diabetes without nephropathy) and healthy controls. Diseased controls were matched to cases by age. Diagnosis of type 2 diabetes was based on the American Diabetes Association criteria of 2003 [26]. Type 2 diabetes with nephropathy was diagnosed on the basis of an overt albuminuria, urinary albumin excretion >300 mg/24 h (>200 µg/min; representing persistent albuminuria) with or without elevated serum creatinine levels (serum creatinine >1.3 mg/dl), determined in at least two separate occasions three months apart from one another, and in the absence of clinical or radiological evidence of non-diabetic renal disease. Infection was excluded by previous urine dipstick tests. Moderately increased albuminuria, formerly called microalbuminuria, i.e., urinary albumin excretion 30–300 mg/24 h (20–200 μg/min), was not categorized as diabetic nephropathy. Existence of arterial hypertension or cardiovascular disease and the glycosylated hemoglobin (HbA1c %) were registered. Blood samples for biochemical measurements and DNA extraction was taken from each individual.

#### Genotyping

Genomic DNA was extracted from peripheral blood leukocytes using a salting out method. Based on Hapmap data for CEU population (Release 27, Phase II + III, Feb09, on NCBI B36 assembly, dbSNP b126) tag single nucleotide polymorphisms (SNPs) across *SLC2A1* (spanning from 42925375 to 42959176, overall 33.802Kbp, on chromosome 1p34.2) were selected using the tagger algorithm (http://www.broadinstitute.org/mpg/tagger/) with a pair-wise approach, an *r*^2^ cut-off of ≥0.8 and a minor allele frequency >0.05. A total of 14 tag SNPs in three distinct gene regions were retrieved (rs12407920 C/T, rs2297976 G/T, rs710221 G/A, rs2086856 A/G, rs12130264 C/T, rs841847 C/T, rs841853 C/A, rs3729548 C/T, rs841855 G/A, rs3768029 C/T, rs12071418 C/G, rs3820549 C/G, rs3820546 G/A, rs11537641 G/A). Concretely, the tag SNPs were located in the intron 1 between exons 1 and 2 (rs12407920, rs2297976, rs710221, rs2086856) and in the intron 2 between exons 2–3 (rs12130264, rs841847, rs841853, rs3729548, rs841855, rs3768029, rs12071418, rs3820549, rs3820546) as well as in the exon 7 (rs11537641).

Genotyping was performed with TaqMan allele specific discrimination assays on an ABI PRISM® 7900 Sequence Detection System (Foster City, CA, USA) and analyzed with SDS software (Applied Biosystems, Foster City, USA). At least 10% of the samples were selected randomly for repeated genotyping, as an internal control. Genotyping was performed by laboratory personnel blinded to clinical status.

#### Data analysis

The data for continuous variables were expressed as mean value and standard deviation [mean ± SD] and data for categorical variables as count (or ratio) and percentage [*n* (%)]. The normality of continuous variables was tested by the Kolmogorov–Smirnov test. Pair-wise comparisons of continuous variables were performed with the *t*-test or the Mann–Whitney *U* test for unpaired data, as appropriate. The frequencies of categorical variables were compared by means of the *χ*^2^ test or the Fisher’s exact test.

The association between genotype distribution and disease progression (i.e., disease progression to diabetic nephropathy) was examined using the generalized linear odds ratio (OR_G_) [21,22]. The OR_G_ expresses the probability of a subject being more diseased relative to the probability of being less diseased, given that diseased subjects have higher mutational load. Explicitly, the OR_G_ shows how many cases/healthy-controls pairs exist in the study for which the cases have larger mutational load relative to the number of pairs for which the healthy controls have the larger mutational load; alternatively, OR_G_ indicates whether the mutational load of a variant is implicating in disease susceptibility [21,22]. The association between genotype distribution and the disease status (i.e., healthy controls, diseased controls and cases) was additionally tested using the *χ*^2^ test.

For the investigation of the susceptibility to type 2 diabetes leading to nephropathy the co-dominant and additive inheritance models of cases were compared to healthy controls using univariate logistic regression. The magnitude of associations was expressed in terms of odds ratios (ORs) unadjusted and adjusted for age and gender with the corresponding 95% confidence interval (CI). These two inheritance models were selected since they are orthogonal [23,24]. From the respective ORs, we calculated the degree of dominance index (h-index) as an estimate for the mode of inheritance [24,25].

In healthy controls, deviation of the genotype distribution from the Hardy–Weinberg equilibrium (HWE) and existence of linkage disequilibrium (LD) between polymorphisms were evaluated using exact tests according to Weir [27,28]. A result was considered to be statistically significant when *p* < .05.

Genotype distribution and the respective unadjusted and adjusted ORs were calculated using IBM® SPSS® Statistics Version 21 (IBM Corp.©, Release 21.0.0.1, 2012, NY, USA). HWE and LD were tested using the Genetic Data Analysis (GDA) software [27,29]. The haplotype frequencies were estimated and compared by SHEsis [30,31]. OR_G_ was calculated using ORGGASMA (http://biomath.med.uth.gr) [22].

##### Meta-analysis

#### Identification and eligibility of relevant studies

In meta-analysis, the published genetic association studies (GAS) regarding variants of *SLC2A1* gene were searched using HuGE Phenopedia (last update in January 2017), the NHGRI Catalog of Published Genome-Wide Association Studies (http://www.genome.gov/gwastudies/) regarding the disease term ‘diabetic nephropathies’ and PubMed with search terms such as ‘diabetic nephropathy’ AND ‘association’ AND (‘gene symbol’ OR ‘gene name’) (accessed on 18 January 2016). All included studies were published in English. We also persused articles from GWAS in HuGE Publit. Finally, relevant meta-analyses and references of the eligible articles were retrieved to identify articles not indexed in PubMed or HuGE Navigator.

The eligible studies should involve cases with persistent micro/macroalbuminuria with or without diabetic retinopathy, diseased controls with diabetes and normoalbuminuria or normal renal function and/or healthy controls. They should provide full genotypic data either genotype counts or allele frequencies and include human subjects. The diabetes could be either T1DM or T2DM.

We did not include in meta-analysis studies investigating disease progression, severity, phenotype modification, response to treatment or survival. Case reports, editorials, reviews and studies with other study designs, such as linkage studies, were also excluded. The eligibility of the articles was assessed independently by two investigators (MT, EZ), the results were compared and any disagreements were resolved by reaching a consensus.

#### Data extraction

From each article information regarding first author, year of publication, ethnicity, the PMID, type of diabetes, country and the phenotype was extracted. For cases and controls, we recorded their number, duration of diabetes, the selection criteria and the implementation of matching criteria. With regard to the genotypic data, we extracted, if available, the full genotype counts or allele frequencies.

#### Data synthesis and analysis

The association between genotype distribution and diabetic nephropathy was examined using the generalized linear odds ratio (OR_G_) [21,22]. The threshold for meta-analysis was the presence of 2 studies per variant. The associations are presented with generalized odds ratios with corresponding 95% confidence intervals using the random effect model. We tested for between-study heterogeneity with Cochran’s Q statistic (considered significant at *p* < .10) and assessed its extent with the I^2^ statistic, which is independent of the number of studies in the meta-analysis and takes values between 0 and 100%, with higher values denoting a greater degree of heterogeneity [32,33]. OR_G_ was calculated using ORGGASMA (http://biomath.med.uth.gr) [22].

For each study, we examined if controls confronted with Hardy–Weinberg equilibrium (HWE) predicted genotypes using Fisher’s exact test. We also tested for ‘small-study effect’ with the Egger test [34].

We also performed subgroup analyses with regard to diabetes type (T1DM or T2DM) and ethnicity (Caucasians versus non-Caucasians), as well as a sensitivity analysis excluding the data of the present association study.

## Results

### Association analysis

#### Clinical profile of participants

The cohort consisted of 197 cases (patients with type 2 diabetes and nephropathy), 155 diseased controls (patients with type 2 diabetes without nephropathy) and 246 healthy controls. All participants were Caucasians of Greek origin. Table 1 shows the demographic and clinical characteristics. Among 197 cases with type 2 diabetes and nephropathy, 11 were under chronic renal replacement therapy. The distribution of age was as follows: above 60 years old were 165 cases (84%), 133 diseased controls (86%) and 223 healthy controls (91%). In 86% of the cases and 79% of diseased controls, the diabetes duration was more than 10 years.

**Table 1.** Clinical profiles of the study-cohort.

Parameters	Case-control study population groups (*n* = 498)						
	HC	DM	*p* Value*		DM-DN	DM + DN	*p* Value*
*N*	246	352	n.a.		155	197	n.a.
Sex [m; *n* (%)]	136 (55.3)	181 (51.4)	.361		74 (47.7)	107 (54.3)	.238
Age (years)	71 ± 9.2	68 ± 8.9	<.001		68 ± 9.1	69 ± 8.8	.427
DM duration (years)	n.a.	16.3 ± 8.0	n.a.		15.7 ± 8.3	16.8 ± 7.8	.508
HbA1c (%)	n.d.	7.35 ± 1.31	n.a.		7.20 ± 1.34	7.47 ± 1.29	.019
Insulin treatment (%)	n.a.	105 (29.8)	n.a.		50 (32.3)	55 (27.9)	.412
Hypertension (%)	0	224 (63.6)	<.001		98 (63.2)	126 (63.9)	.911
Cardiovascular disease (%)	0	110 (31.3)	<.001		41 (26.5)	69 (35.0)	.105
Creatinine (mg/dl)	0.77 ± 0.15	1.46 ± 1.37	<.001		0.90 ± 0.18	1.84 ± 1.67	<.001
Urea (mg/dl)	30 ± 7.9	59 ± 34	<.001		42 ± 13.6	71 ± 38.3	<.001
Albuminuria (mg/d)	n.d.	470 ± 856	n.a.		43.9 ± 53.3	782 ± 1019	<.001
Proteinuria (mg/d)	n.d.	788 ± 1468	n.a.		105 ± 80.0	788 ± 1468	<.001

Clinical profiles of the study-cohort, consisting of 195 cases (i.e., T2DM-nephropathy; DM + DN), 157 diseased controls (i.e., T2DM without nephropathy; DM-DN) and 246 healthy controls (HC). Continuous data are given as mean and standard deviation [x ± SD] and categorical data as count and percentage [*n* (%)].

* *p* Values were calculated by the Mann–Whitney *U* test for continuous variables or the *χ*^2^ test for categorical variables as appropriate.

#### Disease progression

The genotype distributions of the 14 variants in cases, diseased controls and healthy controls, and the respective OR_G_, are shown in Table 2. The healthy controls were conformed to HWE for all variants (*p* ≥ .05). There was a significant association between disease progression and genotype distribution of certain *SLC2A1* variants (rs12407920 C/T, rs841847 C/T and rs841853 C/A). The model-free approach (OR_G_) produced significant results for these very variants, indicating that the risk of disease progression is related to the mutational load of these variants (i.e., diseased subjects have higher mutational load than healthy controls). In addition, the OR_G_ for variant rs3729548 C/T was significant [OR_G_=0.78 (0.62–0.98)] indicating a protective role of allele T of rs3729548 for disease progression to nephropathy (Table 2).

**Table 2.** Distribution of genotypes of SLC2A1 variants.

Variant	Genotype	DM + DN *N* (%)	DM-DN *N* (%)	HC *N* (%)	*p Value	**OR_G_ (95% CI)
rs12407920	C C	154 (79.4)	124 (80.5)	212 (88.0)	**.044**	**1.55 (1.09–2.19)**
	C T	38 (19.6)	30 (19.5)	26 (10.8)		
	T T	2 (1.0)	0 (0)	3 (1.2)		
rs2297976	G G	122 (63.9)	96 (64.0)	153 (63.2)	.973	0.99 (0.76–1.29)
	G T	62 (32.5)	48 (32.0)	82 (33.9)		
	T T	7 (3.7)	6 (4.0)	7 (2.9)		
rs710221	G G	66 (34.6)	49 (33.3)	77 (32.9)	.930	0.98 (0.77–1.24)
	G A	89 (46.6)	74 (50.3)	118 (50.4)		
	A A	36 (18.8)	24 (16.3)	39 (16.7)		
rs2086856	A A	86 (44.8)	73 (48.7)	111 (46.6)	.369	1.11 (0.87–1.41)
	A G	80 (41.7)	64 (42.7)	108 (45.4)		
	G G	26 (13.5)	13 (8.7)	19 (8.0)		
rs12130264	C C	176 (90.3)	136 (89.5)	209 (87.1)	.492	0.80 (0.52–1.23)
	C T	17 (8.7)	16 (10.5)	30 (12.5)		
	C C	2 (1.0)	0 (0.0)	1 (0.4)		
rs841847	C C	86 (44.8)	71 (46.7)	147 (60.2)	**.015**	**1.49 (1.16–1.89)**
	C T	87 (45.3)	67 (44.1)	79 (32.4)		
	T T	19 (9.9)	14 (9.2)	18 (7.4)		
rs841853	C C	70 (36.3)	61 (41.2)	126 (52.3)	**.018**	**1.46 (1.15–1.86)**
	C A	99 (51.3)	69 (46.6)	91 (37.8)		
	A A	24 (12.4)	18 (12.2)	24 (10.0)		
rs3729548	C C	71 (36.8)	48 (32.9)	72 (30.0)	.240	**0.78 (0.62–0.98)**
	C T	94 (48.7)	69 (47.3)	113 (47.1)		
	T T	28 (14.5)	29 (19.9)	55 (22.9)		
rs841855	G G	129 (68.3)	113 (75.8)	169 (70.4)	.509	1.05 (0.79–1.41)
	G A	55 (29.1)	31 (20.8)	65 (27.1)		
	A A	5 (2.6)	5 (3.4)	6 (2.5)		
rs3768029	C C	54 (28.6)	32 (21.3)	65 (26.6)	.213	0.86 (0.68–1.08)
	C T	100 (52.9)	82 (54.7)	115 (47.1)		
	T T	35 (18.5)	36 (24.0)	64 (26.2)		
rs12071418	C C	189 (97.9)	147 (96.1)	231 (96.7)	.583	0.76 (0.36–1.58)
	C G	4 (2.1)	6 (3.9)	8 (3.3)		
	G G	0 (0.0)	0 (0.0)	0 (0.0)		
rs3820549	C C	102 (53.1)	86 (56.2)	124 (50.8)	.842	0.94 (0.74–1.20)
	C G	72 (37.5)	56 (36.6)	98 (40.2)		
	G G	18 (9.4)	11 (7.2)	22 (9.0)		
rs3820546	G G	49 (25.8)	42 (27.6)	70 (29.0)	.362	0.96 (0.77–1.20)
	G A	107 (56.3)	75 (49.3)	113 (46.9)		
	A A	34 (17.9)	35 (23.0)	58 (24.1)		
rs11537641	G G	131 (67.9)	107 (70.9)	159 (66.0)	.484	0.91 (0.69–1.20)
	G A	58 (30.1)	39 (25.8)	70 (29.0)		
	A A	4 (2.1)	5 (3.3)	12 (5.0)		

Distribution of genotypes of *SLC2A1* variants among cases with T2DM-nephropathy (T2DM-nephropathy; DM + DN), diseased (T2DM without nephropathy; DM-DN) and healthy (HC) control subjects. *χ^2^*p* Values and **the generalized odds ratio (OR_G_) with respective 95% confidence intervals (95% CI) calculated for testing the association between genotype distribution of each variant and disease progression. OR_G_ and *p* values are in bold in case of statistical significance.

#### Type 2 diabetes leading to nephropathy

##### Single-locus analysis

Table 3 shows the association results for type 2 diabetes leading to nephropathy. Analysis of the co-dominant and the additive inheritance model shown indicates that certain variants were associated with the risk of type 2 diabetes leading to nephropathy. Significant results were derived for the co-dominant inheritance model of the variants rs12407920 C/T [OR = 2.01 (1.17–3.45)], rs841847 C/T [OR = 1.73 (1.17–2.56)] and rs841853 C/A [OR = 1.74 (1.18–2.55)] as well as for the additive inheritance model of the variant rs3729548 C/T [OR = 0.52 (0.29–0.90)]. The mode of inheritance for the variants rs12407920 C/T, rs841847 C/T and rs841853 C/A was ‘dominance of each minor allele’and for the variant rs3729548 C/T was ‘non-dominance’. Therefore, for the variants rs12407920 C/T (*h* = 8.37), rs841847 C/T (*h* = 0.93) and rs841853 C/A (*h* = 0.94), the mode of inheritance is ‘dominance of each minor allele’, indicating that the homozygous for the minor allele has a greater risk of being diabetic with nephropathy than the homozygous for the frequent allele, and that the heterozygote has a risk of diabetes leading to nephropathy closer to the homozygote for the minor allele than to the midpoint between the two homozygotes. The mode of inheritance attributed to the variant rs3729548 C/T is ’non-dominance’ (*h* = 0.10), indicating that the heterozygote CT has a risk of being diseased that lies in the middle of the risk-protected CC and risk-exposed TT homozygous genotypes (Table 4).

**Table 3.** Association between SLC2A1 gene variants and T2DM- nephropathy for the additive and co-dominant models.

Variant	Genetic model	OR (95% CI)	*p-Value	ORadj. (95% CI)	#*p*-Value
rs12407920 C/T	Additive	0.92 (0.15–5.56)	0.926	0.69 (0.09-5.02)	.715
	Co-dominant	**2.01 (1.17–3.45)**	**0.010**	**1.98 (1.14–3.43)**	**.015**
rs2297976 G/T	Additive	1.25 (0.43–3.67)	0.679	1.17 (0.39–3.47)	.778
	Co-dominant	0.94 (0.63–1.40)	0.755	0.92 (0.61–1.38)	.677
rs710221 G/A	Additive	1.08 (0.61–1,88)	0.795	1.01 (0.56–1.81)	.987
	Co-dominant	0.86 (0.58–1.26)	0.432	0.85 (0.58–1.26)	.425
rs2086856 A/G	Additive	1.77 (0.92–3.40)	0.089	1.85 (0.94–3.65)	.075
	Co-dominant	0.86 (0.59–1.26)	0.441	0.85 (0.58–1.26)	.452
rs12130264 C/T	Additive	2.37 (0.21–26,41)	0.482	2.81 (0.25–31,79)	.403
	Co-dominant	0.67 (0.36 1.25)	0.209	0.72 (0.38–1.38)	.327
rs841847 C/T	Additive	1.80 (0.90–3.62)	0.097	1.77 (0.84–3.70)	.131
	Co-dominant	**1.73 (1.17–2.56)**	**0.006**	**1.73 (1.16–2.58)**	**.007**
rs841853 C/A	Additive	1.80 (0.95–3.40)	0.070	1.85 (0.94–3.61)	.073
	Co-dominant	**1.74 (1.18–2.55)**	**0.005**	**1.75 (1.18–2.58)**	**.005**
rs3729548 C/T	Additive	**0.52 (0.29–0.90)**	**0.021**	**0.50 (0.28–0.92)**	**.025**
	Co-dominant	1.07 (0.73–1.56)	0.737	1.06 (0.72–1.56)	.778
rs841855 G/A	Additive	1.09 (0.33–3,66)	0.887	1.04 (0.31–3.55)	.948
	Co-dominant	1.10 (0.72–1.69)	0.644	1.12 (0.73–1.73)	.608
rs3768029 C/T	Additive	0.66 (0.38–1.14)	0.135	0.65 (0.37–1,16)	.146
	Co-dominant	1.26 (0.86–1.84)	0.233	1.29 (0.87–1,90)	.202
rs12071418 C/G	Additive	**n.a.	**n.a.	**n.a.	**n.a.
	Co-dominant	0.61 (0.18–2.06)	0.427	0.59 (0.17–2.01)	.396
rs3820549 C/G	Additive	0.99 (0.51–1.95)	0.988	0.99 (0.50–1.99)	.987
	Co-dominant	0.89 (0.61–1,32)	0.571	0.88 (0.59–1.30)	.517
rs3820546 G/A	Additive	0.84 (0.48–1.46)	0.534	0.85 (0.48–1,50)	.577
	Co-dominant	1.46 (0.99–2.14)	0.052	1.35 (0.92–2.00)	.126
rs11537641 G/A	Additive	0.40 (0.13–1.28)	0.125	0.36 (0.11–1,17)	.089
	Co-dominant	1.05 (0.69–1.59)	0.819	1.03 (0.68–1.58)	.874

Odds Ratio (OR) and the corresponding 95% Confidence Intervals (CI) for testing the association between the SLC2A1 gene variants and T2DM-nephropathy for the additive and co-dominant models. The ORs adjusted for age and sex calculated by univariate logistic regression are also shown. In all variants, the ORs are calculated considering the heterozygote as the risk genotype for the co-dominant model and the minor allele as the risk allele for the additive model.

* *χ*^2^*p* Values.

# Multivariate logistic regression *p* values.

** Not applicable, because there are no G allele homozygotes. OR and *p* values are in bold in case of statistical significance.

**Table 4.** The degree of dominance index (h-index).

Variant	h-Index	Mode of inheritance
rs12407920 C/T	8.37	Dominance of allele T
rs841847 C/T	0.93	Dominance of allele T
rs841853 C/A	0.94	Dominance of allele A
rs3729548 C/T	0.10	None-dominance (additiveness)

The degree of dominance index (h-index) as an estimate for the mode of inheritance, calculated on the basis of unadjusted odds ratio (OR) values and the respective mode of inheritance for all *SLC2A1* variants with a significant association to type 2 diabetes leading to nephropathy, found for the additive and co-dominant inheritance models.

##### Linkage disequlibrium analysis

Table 5 shows the D′, *r*^2^ and *p* values for testing linkage disequilibrium (LD) between pairs of *SCL2A1* variants for patients with diabetes leading to nephropathy (cases) and healthy controls. In cases 107 out of 182 (59%) and in healthy controls 120 out of 182 (66%) pairs of variants tested were in LD (*p* < .05). In 11 of the 14 *SLC2A1* variants above 50% of variant pairs were in LD in both populations (cases and healthy controls). Especially, variant rs12071418 C/G was not in LD with any other variant in cases, whereas it was in LD with only 3 other variants (rs2297976 G/T, rs3820549 C/G, rs11537641 G/A) in controls. Moreover, omitting the rs12071418 C/G variant pairs, lead to 63% of pairs being in LD in cases and 69% in healthy controls, respectively.

**Table 5.** Estimates (D′, *r*^2^ and *p* values) of linkage disequilibrium (LD) between pairs of *SLC2A1* variants.

Variant	LD	rs2297976	rs710221	rs2086856	rs12130264	rs841847	rs841853	rs3729548	rs841855	rs3768029	rs12071418	rs3820549	rs3820546	rs11537641
rs12407920	D′	0.99 (0.99)	1.00 (0.99)	1.00 (1.00)	0.04 (0.59)	0.31 (0.49)	0.89 (0.83)	0.85 (0.89)	0.99 (0.99)	0.04 (0.22)	0.04 (1.00)	0.19 (0.65)	0.41 (0.57)	0.69 (0.99)
	*r*^2^	0.03 (0.02)	0.09 (0.05)	0.21 (0.15)	0.00 (0.00)	0.02 (0.06)	0.16 (0.12)	0.05 (0.05)	0.03 (0.01)	0.00 (0.00)	0.00 (0.00)	0.00 (0.01)	0.02 (0.02)	0.01 (0.02)
	*p*	.03 (.09)	<.01	<.01	.18 (.29)	.1 (<.01)	<.01	.12 (<.01)	.45 (.15)	.26 (.29)	1.00 (.18)	.40 (.14)	.20 (.01)	.28 (.06)
rs2297976	D′	–	1.00 (1.00)	0.99 (0.99)	1.00 (0.99)	0.76 (0.63)	0.77 (0.64)	0.87 (0.97)	0.99 (0.53)	0.90 (0.84)	0.58 (0.80)	0.65 (0.10)	0.04 (0.24)	0.17 (0.04)
	*r*^2^	–	0.35 (0.33)	0.13 (0.11)	0.01 (0.02)	0.30 (0.32)	0.24 (0.25)	0.12 (0.19)	0.05 (0.01)	0.17 (0.18)	0.01 (0.04)	0.04 (0.01)	0.00 (0.01)	0.00 (0.00)
	*p*	–	<.01	<.01	<.01 (.09)	<.01	<.01	<.01	.26 (.80	<.01	.38 (.02)	.19 (.61)	.45 (.30)	.97 (.21)
rs710221	D′	–	–	0.38 (0.51)	1.00 (0.99)	0.18 (0.26)	0.09 (0.21)	0.88 (0.88)	0.85 (0.93)	0.76 (0.66)	0.26 (0.99)	0.31 (0.59)	0.42 (0.43)	0.89 (0.87)
	*r*^2^	–	–	0.10 (0.16)	0.04 (0.05)	0.02 (0.03)	0.00 (0.02)	0.37 (0.49)	0.21 (0.21)	0.35 (0.32)	0.00 (0.02)	0.05 (0.19)	0.11 (0.12)	0.21 (0.25)
	*p*	–	–	<.01	<.01	.38 (.04)	.68 (.13)	<.01	<.01	<.01	.73 (.09)	.02 (<.01)	<.01	<.01
rs2086856	D′	–	–	–	0.99 (0.99)	0.47 (0.29)	0.13 (0.05)	0.81 (0.79)	0.85 (0.85)	0.43 (0.46)	0.04 (0.63)	0.40 (0.37)	0.58 (0.65)	0.70 (0.54)
	*r*^2^	–	–	–	0.03 (0.03)	0.05 (0.01)	0.00 (0.00)	0.21 (0.24)	0.30 (0.31)	0.08 (0.10)	0.00 (0.00)	0.12 (0.12)	0.15 (0.18)	0.19 (0.16)
	*p*	–	–	–	.04 (.28)	.02 (.03)	.48 (.47)	<.01	<.01	.01 (<.01)	.57 (.89)	<.01	<.01	<.01
rs12130264	D′	–	–	–	–	0.16 (0.99)	0.32 (0.81)	0.72 (0.91)	0.97 (0.99)	0.47 (0.45)	1.00 (0.99)	0.99 (0.84)	0.26 (0.72)	0.96 (0.99)
	*r*^2^	–	–	–	–	0.00 (0.02)	0.00 (0.02)	0.04 (0.07)	0.01 (0.01)	0.01 (0.01)	0.00 (0.00)	0.02 (0.02)	0.01 (0.04)	0.01 (0.02)
	*p*	–	–	–	–	.10 (.13)	.06 (.07)	<.01	.28 (.65)	.12 (.09)	.17 (.86)	.10 (.03)	.01 (.02)	.22 (.23)
rs841847	D′	–	–	–	–	–	1.00 (1.00)	1.00 (1.00)	0.99 (0.99)	0.93 (0.95)	1.00 (0.96)	0.33 (0.35)	0.24 (0.40)	0.81 (0.81)
	*r*^2^	–	–	–	–	–	0.78 (0.74)	0.30 (0.26)	0.10 (0.06)	0.34 (0.28)	0.01 (0.01)	0.02 (0.02)	0.02 (0.04)	0.06 (0.05)
	*p*	–	–	–	–	–	<.01	<.01	<.01 (.01)	<.01	.25 (.37)	.23 (.18)	.21 (.00)	.02 (.00)
rs841853	D′	–	–	–	–	–	–	1.00 (1.00)	0.99 (0.99)	0.69 (0.63)	0.99 (0.99)	0.19 (0.13)	0.29 (0.39)	0.85 (0.80)
	*r*^2^	–	–	–	–	–	–	0.39 (0.35)	0.13 ((0.08)	0.24 (0.16)	0.01 (0.01)	0.01 (0.00)	0.04 (0.05)	0.09 (0.06)
	*p*	–	–	–	–	–	–	<.01	<.01	<.01	.36 (.38)	.29 (.46)	.06 (<.01)	<.01
rs3729548	D′	–	–	–	–	–	–	–	1.00 (1.00)	0.94 (0.84)	0.04 (0.99)	0.71 (0.75)	0.63 (0.56)	0.79 (0.89)
	*r*^2^	–	–	–	–	–	–	–	0.13 (0.16)	0.66 (0.61)	0.00 (0.01)	0.12 (0.19)	0.29 (0.29	0.08 (0.17
	*p*	–	–	–	–	–	–	–	<.01	<.01	.65 (.29)	<.01	<.01	<.01
rs841855	D′	–	–	–	–	–	–	–	–	1.00 (1.00)	0.59 (0.08)	0.78 (0.94)	0.72 (0.91)	0.80 (0.85)
	*r*^2^	–	–	–	–	–	–	–	–	0.17 (0.19)	0.00 (0.00)	0.34 (0.42)	0.09 (0.14)	0.60 (0.59)
	*p*	–	–	–	–	–	–	–	–	<.01	1.00 (.84)	<.01	<.01	<.01
rs3768029	D′	–	–	–	–	–	–	–	–	–	0.91 (0.99)	0.52 (0.57)	0.57 (0.59)	0.84 (0.88)
	*r*^2^	–	–	–	–	–	–	–	–	–	0.01 (0.02)	0.09 (0.13)	0.30 (0.32)	0.12 (0.18)
	*p*	–	–	–	–	–	–	–	–	–	.84 (.13)	<.01	<.01	<.01
rs12071418	D′	–	–	–	–	–	–	–	–	–	–	0.99 (1.00)	0.96 (0.99)	0.23 (0.81)
	*r*^2^	–	–	–	–	–	–	–	–	–	–	0.02 (0.04)	0.01 (0.02)	<0.01 (0.05)
	*p*	–	–	–	–	–	–	–	–	–	–	.13 (.01)	.17 (.19)	.59 (.01)
rs3820549	D′	–	–	–	–	–	–	–	–	–	–	–	1.00 (1.00)	0.93 (0.95)
	*r*^2^	–	–	–	–	–	–	–	–	–	–	–	0.33 (0.37)	0.45 (0.54)
	*p*	–	–	–	–	–	–	–	–	–	–	–	<.01	<.01
rs3820546	D′	–	–	–	–	–	–	–	–	–	–	–	–	1.00 (1.00)
	*r*^2^	–	–	–	–	–	–	–	–	–	–	–	–	0.17 (0.22)
	*p*	–	–	–	–	–	–	–	–	–	–	–	–	<.01

Estimates (D′, *r*^2^ and *p* values) of linkage disequilibrium (LD) between pairs of *SLC2A1* variants (SNPs) in patients with type 2 diabetes leading to nephropathy (cases, DN) and in healthy controls (HC). LD estimates of HC are shown in brackets.

##### Analysis of haplotypes

The distribution of the estimated haplotype frequencies of the 14 *SLC2A1* variants (rs12407920 C/T, rs2297976 G/T, rs710221 G/A, rs2086856 A/G, rs12130264 C/T, rs841847 C/T, rs841853 C/A, rs3729548 C/T, rs841855 G/A, rs3768029 C/T, rs12071418 C/G, rs3820549 C/G, rs3820546 G/A, rs11537641 G/A) for cases and healthy controls is presented in Table 6. The overall difference between cases and healthy controls was not significant (*p* = .132). In the analysis of the individual haplotypes, however, C-G-G-A-T-C-C-T-G-T-C-C-A-G derived significant results [*p* = .014; OR = 0.248 (0.075–0.817)]. This haplotype may confer protection for type 2 diabetes leading to nephropathy, as alleles, which were shown to increase the risk of diabetes leading to nephropathy (i.e., allele T of rs12407920 C/T, allele T of rs841847 C/T and allele A of rs841853 C/A), are all missing in the haplotype, whereas allele T of rs3729548 C/T, which seems to act protectively, is included.

**Table 6.** Estimated haplotype frequencies for the 14 *SLC2A1* variants.

	Estimated frequencies			*χ*^2^	Global
Haplotypes of SNPs 1–14*	DM + DN	HC	Odds ratio (95%CI)	*p*-Value	*p*-Value
C G A G C C C C A C C G G A	0.127	0.121	1.009 (0.638–0.597)	.969	.132
C T A A C T A C G C C C A G	0.074	0.067	1.063 (0.594–0.902)	.838	
C G G A C C C T G T C C A G	0.285	0.290	0.908 (0.637–0.292)	.591	
C G G A T C C T G T C C A G	0.010	0.038	**0.248 (0.075–0.817)**	**.014**	
C T A A C T A C G C C C G G	0.097	0.060	1.633 (0.927–0.875)	.087	
C G G A C T A C G C C C G G	0.031	0.015	2.067 (0.726–0.888)	.165	
T G G G C T A C G C C C G G	0.032	0.041	0.732 (0.330–0.627)	.443	
C G G A C C C T G T C C G G	0.039	0.048	0.773 (0.371–0.610)	.491	
C G G A C T A C G C C G G G	0.044	0.029	1.481 (0.667–0.288)	.332	

Estimated haplotype frequencies for the 14 *SLC2A1* variants (SNPs 1–14: rs12407920 C/T, rs2297976 G/T, rs710221 G/A, rs2086856 A/G, rs12130264 C/T, rs841847 C/T, rs841853 C/A, rs3729548 C/T, rs841855 G/A, rs3768029 C/T, rs12071418 C/G, rs3820549 C/G, rs3820546 G/A, rs11537641 G/A) in cases (T2DM-nephropathy; DM + DN) and in healthy controls (HC). The *p* values for comparison between cases and HC of the frequencies of each haplotype and the global *p* values for testing the overall difference in haplotype frequencies are shown.

* Only haplotypes with a frequency of >0.03 in either cases (DM + DN) or controls (HC) are shown. OR and *p* values are in bold in case of statistical significance.

### Meta-analysis

**Figure 1.**
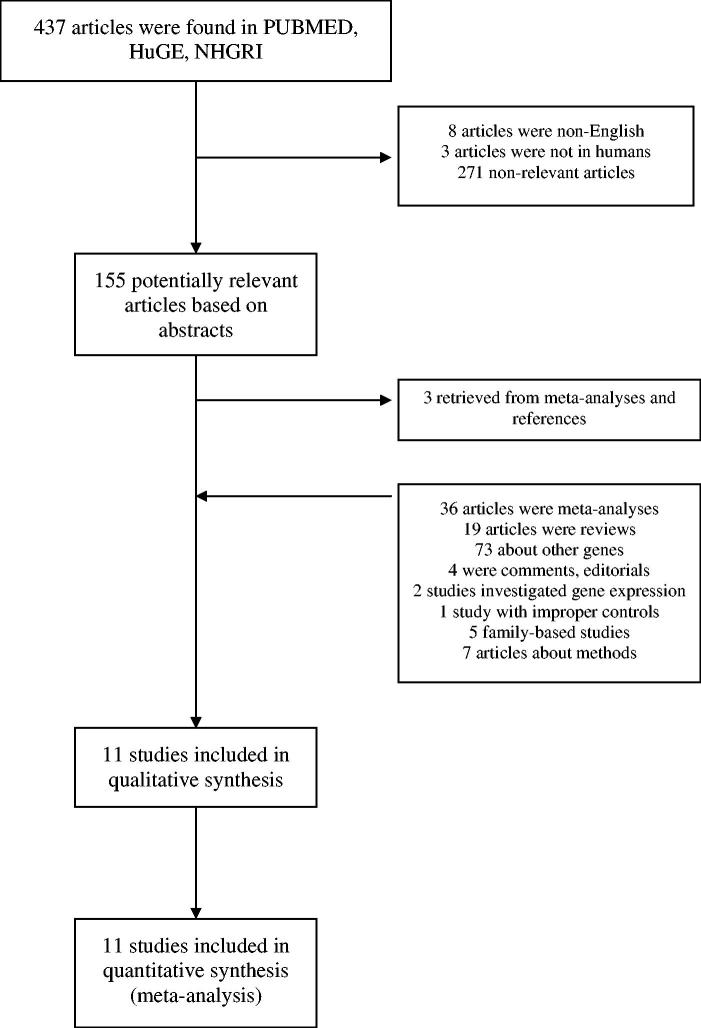
Flowchart showing how studies were selected for meta-analysis.

**Figure 2.**
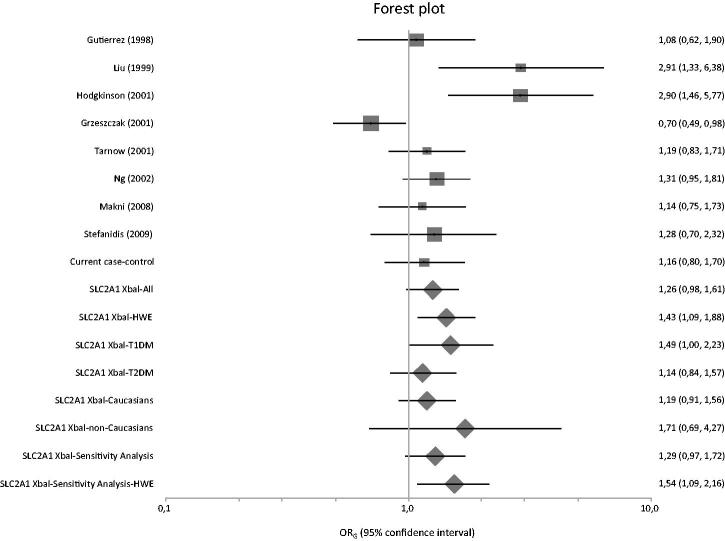
Forest plot presenting results of individual studies and pooled estimates from both main and subgroup meta-analyses between diseased controls versus cases.

**Figure 3.**
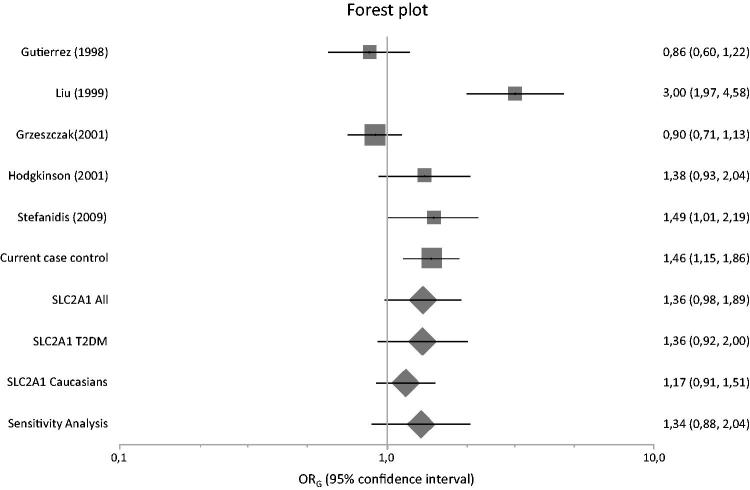
Forest plot presenting results of individual studies and pooled estimates from both main and subgroup meta-analyses between healthy controls versus diseased controls versus cases.

**Figure 4.**
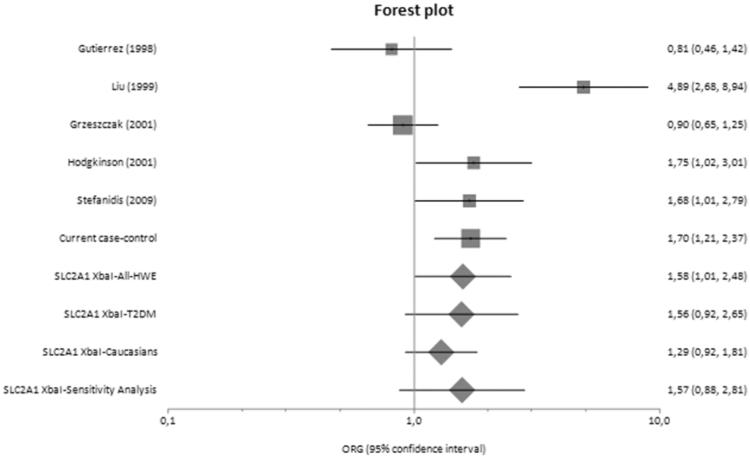
Forest plot presenting results of individual studies and pooled estimates from both main and subgroup meta-analyses between healthy controls versus cases.

Figure 1 presents a flowchart of retrieved and excluded articles. The characteristics of each study are shown in Table 7. Across all available studies examining *SLC2A1* variants, 8 genetic variants were studied (XbaI SNP, HaeIII SNP, Enhancer-1 SNP, Enhancer-2 SNP 1, Enhancer-2 SNP 2, Enhancer-3 SNP, HpyCH4V and rs3820589). Out of the aforementioned variants, only five variants examined in two studies or more and so meta-analyzed (XbaI SNP, HaeIII SNP, Enhancer-2 SNP 1, Enhancer-2 SNP 2 and HpyCH4V). Only XbaI SNP produced significant results in analysis using diseased controls versus cases and healthy controls versus cases giving a summary OR_G_ of 1.428 (1.086, 1.877) and 1.581 (1.007, 2.482), respectively (Table 8). The studies comprised 1812 cases, 1763 diseased controls and 949 healthy controls and they were published between 1998 and 2015 [10,35–44]. Figures 2–4 are forest plot representations of variant rs841853.

**Table 7.** Characteristics of studies included in meta-analysis.

Variant	References	Ethnicity	PMID	DM	Trait	*N*	Selection criteria	*N*	Selection criteria	*N*	Selection criteria	HWE HT	HWE DC	Analyses
SLC2A1 rs841853	[10]	Caucasians	19822956	T2DM	DN	92	Pers. albuminuria	56	DM >15 y, pers. norm/ria matched for age, BMI	92	Non-diabetics matched for age, BMI		No	DC-C, HT-DC-C, HT-C
	[36]	Africans (Tunisia)	18821326	T2DM	DN	126	Pers. albuminuria, retinopathy	273	DM >10 y, norm/ria				No	DC-C
	[37]	Caucasians	12086959	T1DM	DN	262	Pers. macr/ria or d. ESRD	230	Pers. norm/ria , DM ≥15 y					DC-C
	[38]	Caucasians	11231353	T1DM	DN	70	Pers. proteinuria, retinopathy, DM ≥10 y	44	DM≥ 20 y, pers. norm/ria	104	Non-diabetics			DC-C, HT-DC-C, HT-C
	[39]	Caucasians	11477169	T1DM	DN	199	Pers. macr/ria , retinopathy	192	Pers. norm/ria matched for gender, age, DM duration					DC-C
	[40]	E. Asians	10231446	T2DM	DN	64	Pers. albuminuria or proteinuria with or without impaired renal function	45	Only diabetics	124	Non-diabetics			DC-C, HT-DC-C, HT-C
	[41]	Caucasians	11168944	T2DM	DN	282	Pers. micr/ria/proteinuria/CRF	162	Pers. norm/ria, DM ≥10 y	194	Non-diabetics		No	DC-C, HT-DC-C, HT-C
	[42]	Caucasians	9789717	T2DM	DN	60	Pers. micro/macroalbuminuria	100	Pers. norm/ria matched for gender, age, BMI, DM duration and HbA1c and lipidic profile	90	Non-diabetics			DC-C, HT-DC-C, HT-C
														
SLC2A1 rs1385129	[43]	Caucasians-Brazilians	25701507	T1DM	DN	203	Pers. micro/macroalbuminuria	249	Pers. norm/ria and serum Cr.<1.7 mg/dl					DC-C
	[44]	Asians	26337659	T2DM	DN	126	Pers. micr/ria	150	Pers. norm/ria				No	DC-C
	[36]	Africans (Tunisia)	18821326	T2DM	DN	126	Pers. albuminuria, retinopathy	273	DM >10 y, norm/ria					DC-C
	[37]	Caucasians	12086959	T1DM	DN	262	Pers. macr/ria or d. ESRD	230	Pers. norm/ria , DM duration ≥15 y					DC-C
														
SLC2A1 rs841847	[36]	Africans (Tunisia)	18821326	T2DM	DN	126	Pers. albuminuria, retinopathy	273	DM >10 y, norm/ria					DC-C
	[43]	Caucasians-Brazilians	25701507	T1DM	DN	203	Pers. micro/macroalbuminuria	249	Pers. norm/ria and serum Cr.<1.7 mg/dl					DC-C
	[37]	Caucasians	12086959	T1DM	DN	262	Pers. macr/ria or d. ESRD	230	Pers. norm/ria, DM ≥15 y					DC-C
SLC2A1 rs841848	[36]	Africans (Tunisia)	18821326	T2DM	DN	126	Pers. albuminuria, retinopathy	273	DM >10 y, norm/ria					DC-C
	[43]	Caucasians-Brazilians	25701507	T1DM	DN	203	Pers. micro/macroalbuminuria	249	Pers. norm/ria and s. Cr.<1.7 mg/dl					DC-C
	[37]	Caucasians	12086959	T1DM	DN	262	Pers. macr/ria or d. ESRD	230	Pers. norm/ria , DM ≥15 y					DC-C
SLC2A1 rs710218	[36]	Africans (Tunisia)	18821326	T2DM	DN	126	Pers. albuminuria, retinopathy	273	DM >10 y, norm/ria				No	DC-C
	[38]	Caucasians	15745834	T1DM	DN	131	DM ≥10 y, pers. proteinuria, diabetic retinopathy	72	Uncomplicated, DM ≥20 y without retinopathy and proteinuria	99	Non-diabetics			DC-C
								35	DM <10 y without retinopathy, proteinuria or overt neuropathy					

**Table 8.** Results from meta-analyses based on genotype counts.

Diseased controls versus cases										
Gene	Variant	RS	Studies (*n*)	Cases/Controls (*n*)	RE OR_G_	95% LL	95% UL	I^2^(%)	P_Q_	P_E_
SLC2A1	XbaI(+)>XbaI(−)	rs841853	9	1311/1226	1.26	0.98	1.61	62.31	0.01	0.04
SLC2A1	All in HWE		6	811/735	1.43	1.09	1.88	50.90	0.07	0.08
*Subgroup analyses*										
T1DM	XbaI(+)>XbaI(−)	rs841853	3	494/443	1.49	1.00	2.23	62.13	0.07	0.16
T2DM	XbaI(+)>XbaI(−)	rs841853	6	817/783	1.14	0.84	1.57	60.86	0.03	0.05
Caucasians	XbaI(+)>XbaI(−)	rs841853	7	1121/908	1.19	0.91	1.56	62.17	0.01	0.12
Non-Caucasians	XbaI(+)>XbaI(−)	rs841853	2	190/318	1.71	0.69	4.27	76.70	0.04	na
*Sensitivity analysis*										
Excluding current case-control	XbaI(+)>XbaI(−)	rs841853	8	1118/1078	1.29	0.97	1.72	67.020	0	0.05
	All in HWE		5	618/587	1.54	1.09	2.16	57.70	0.05	0.13
										
SLC2A1	HaeIII SNP	rs1385129	4	716/899	0.74	0.350	1.57	92.05	0	0.18
	All in HWE		3	590/749	1.14	0.73	1.76	74.96	0.02	0.11
										
SLC2A1	Enh2 SNP1 G>A	rs841847	4	783/904	1.48	0.85	2.60	89.27	0	0.13
SLC2A1	All in HWE		4							
*Subgroup analyses*										
T1DM	Enh2 SNP1 G>A	rs841847	2	465/479	1.10	0.87	1.40	0	0.92	na
T2DM	Enh2 SNP1 G>A	rs841847	2	318/425	2.02	0.58	7.00	94.83	0	na
Caucasians	Enh2 SNP1 G>A	rs841847	2	454/382	1.08	0.85	1.39	0	0.96	na
Non-Caucasians	Enh2 SNP1 G>A	rs841847	2	329/522	2.06	0.62	6.86	94.81	0	na
*Sensitivity analysis*										
Excluding current case-control	Enh2 SNP1 G>A	rs841847	3	591/752	1.65	0.78	3.51	92.21	0	0.16
	All in HWE		3							
										
SLC2A1	Enh2 SNP2 C>T	rs841848	3	588/750	1.18	0.95	1.45	0	0.62	0.35
	All in HWE		3							
*Subgroup analyses*										
T1DM	Enh2 SNP2 C>T	rs841848	2	462/477	1.14	0.88	1.48	0	0.37	na
T2DM	Enh2 SNP2 C>T	rs841848	1	na	na	na	na	na	na	na
Caucasians	Enh2 SNP2 C>T	rs841848	2	462/477	1.14	0.88	1.48	0	0.37	na
Non-Caucasians	Enh2 SNP2 C>T	rs841848	1	na	na	na	na	na	na	na
*Sensitivity analysis*										
Excluding current case-control	Enh2 SNP2 C>T	rs841848	na	na	na	na	na	na	na	na
										
SLC2A1	HpyCH4V	rs710218	2	257/380	3.87	0.61	24.38	96.94	0.00	na
	All in HWE		2							
Healthy controls versus diseased controls versus cases										
SLC2A1	XbaI(+)>XbaI(−)	rs841853	6	761/554/845	1.36	0.98	1.89	84.03	0	0.10
SLC2A1	All in HWE		6							
*Subgroup analyses*										
T1DM	XbaI(+)>XbaI(−)	rs841853	1	na	na	na	na	na	na	na
T2DM	XbaI(+)>XbaI(−)	rs841853	5	691/510/741	1.36	0.92	2.00	87.14	0	0.17
Caucasians	XbaI(+)>XbaI(−)	rs841853	5	697/509/721	1.17	0.91	1.51	69.98	0.01	0.21
Non-Caucasians	XbaI(+)>XbaI(−)	rs841853	1	na	na	na	na	na	na	na
*Sensitivity analysis*										
Excluding current case-control	XbaI(+)>XbaI(−)	rs841853	5	568/406/604	1.34	0.88	2.04	86.30	0	0.07
	All in HWE		5							
Healthy controls versus cases										
SLC2A1	XbaI(+)>XbaI(−)	rs841853	6	761/845	1.58	1.01	2.48	83.03	0	0.23
	All in HWE		6							
*Subgroup analyses*										
T1DM	XbaI(+)>XbaI(−)	rs841853	1	na	na	na	na	na	na	na
T2DM	XbaI(+)>XbaI(−)	rs841853	5	691/741	1.56	0.92	2.65	86.15	0	0.28
Caucasians	XbaI(+)>XbaI(−)	rs841853	5	697/721	1.29	0.92	1.81	66.67	0.02	0.42
Non-Caucasians	XbaI(+)>XbaI(−)	rs841853	1	na	na	na	na	na	na	na
*Sensitivity Analysis*	XbaI(+)>XbaI(−)	rs841853	5	568/604	1.568	0.875	2.81	85.765	0	0.15
Excluding current case-control	All in HWE		5							

na: non-applicable.

Regarding the subgroup analyses according to diabetes types and ethnicity, the relevant results were not significant. However, in sensitivity analysis, when the present association study was excluded, the patterns of results changed (Figure 2).

## Discussion

The present study investigated whether *SLC2A1* variants, certain 14 tag SNPs, are associated with the type 2 diabetes disease progression and with the development of type 2 diabetes mellitus leading to nephropathy and also provided the most comprehensive overview assessing for all genetic variants of *SLC2A1* that have been examined in genetic association studies regarding diabetic nephropathy.

Upon examining the association between *SLC2A1* variants and type 2 diabetes leading to nephropathy, we selected as a control population the healthy subjects and not the patients with diabetes type 2 without nephropathy since every participant of the latter population is always a candidate to become a future case with diabetic nephropathy. Moderately increased albuminuria was not categorized as diabetic nephropathy because the diagnosis of diabetic nephropathy cannot be based only on the presence of moderately increased albuminuria. Apart from diabetic nephropathy there are several other causes for moderately increased albuminuria in diabetic patients. In addition, patients with moderately increased albuminuria do not invariably develop nephropathy. The strict selection criteria in our study ensured a relative clear case definition. At the end, only a histological examination would ensure the diagnosis of diabetic nephropathy, however, kidney biopsies are not routinely carried out in diabetes. Among patients with diabetes and nephropathy, who underwent kidney biopsy, the prevalence of diabetic nephropathy was found to be about 73% [45].

The analysis showed that certain variants of *SLC2A1* (rs12407920 C/T, rs841847 C/T, rs841853 C/A and rs3729548 C/T) are involved in disease progression. In addition, these variants are associated with the risk of diabetes leading to nephropathy: significant results were derived for the co-dominant model of the variants rs12407920 C/T [OR = 2.01 (1.17–3.45)], rs841847 C/T [OR = 1.73 (1.17–2.56)] and rs841853 C/A [OR = 1.74 (1.18–2.55)] as well as for the additive model of the variant rs3729548 C/T [OR = 0.52 (0.29–0.90)]. The mode of inheritance for the variants rs12407920 C/T, rs841847 C/T and rs841853 C/A was ‘dominance of each minor allele’ and for the variant rs3729548 C/T was ‘non-dominance’. The frequency of one haplotype (C-G-G-A-T-C-C-T-G-T-C-C-A-G) was significantly different on a comparison between cases and healthy controls [*p* = .014; OR = 0.248 (0.075–0.817)]. This haplotype may confer protection for type 2 diabetes leading to nephropathy, as all the alleles contributing to the risk of diabetes leading to nephropathy (i.e., allele T of rs12407920 C/T, allele T of rs841847 C/T and allele A of rs841853 C/A) are missing in the haplotype, whereas allele T of rs3729548 C/T, which seems to act protectively, is included.

In agreement to our findings, a previous systematic review and meta-analysis of nine genetic association studies in patients with either type 1 or type 2 diabetes found that certain genetic variants in *SLC2A1* (rs1385129, rs841847, rs841848 and rs841853) enhance susceptibility to diabetic nephropathy [46]. The similarity of findings in type 1 and type 2 diabetes mellitus is not surprising. The pathogenesis of diabetic nephropathy is generally the same in all types of diabetes. It is principally related to hyperglycemia and to arterial hypertension. In this context, the putative pathogenic role of the cell-membrane glucose transporter GLUT1 is mainly depending on hyperglycemia, i.e., it is depending on diabetes control and not on diabetes type.

In our study the genetic association was performed in patients with type 2 diabetes of Greek (Caucasian) origin. Subgroup analyses for Caucasians, in the above systematic review, revealed association between diabetic nephropathy due to both type 1 and type 2 diabetes and *SLC2A1* variants [46]. All four studies concerning type 1 diabetes mellitus in the analysis were including only Caucasian populations. There were two studies (out of five) concerning type 2 diabetes mellitus, which included non-Caucasian populations, one in Asians and one in Tunisians, and both showed a positive association between diabetic nephropathy and *SLC2A1* variants. A more recent genetic association study in Brazilian patients with type 1 diabetes mellitus and inadequate blood glucose control showed that another variant of *SLC2A1* (rs3820589) is associated with progression of nephropathy [42]. Along with our findings, these reports clearly implicate a modulating role for SLC2A1 variants in diabetic nephropathy.

However, any genetic association study on the risk for development of diabetic nephropathy in either type of diabetes mellitus might be readily confounded if the genetic factors under investigation are also predisposing to diabetes. This is especially true when healthy controls, i.e., controls without diabetes, are included. *SLC2A1* may not be involved in the pathogenesis of type 1 diabetes but its involvement in type 2 diabetes is plausible according to functional criteria. Nevertheless, according to a study of two populations in the Pacific, the variant rs841853 of SLC2A1 was not predisposing to diabetes type 2. Concretely, the rs841853 alleles frequency was the same (high) in both populations, one Polynesian population with high prevalence for type 2 diabetes and one highland New Guinean population with a notable absence of type 2 diabetes [47]. In contrast, the meta-analysis by Cui et al. (2013) provides strong evidence for Asians and marginal evidence for Caucasians, that the rs841853 variant of *SLC2A1* may confer increased susceptibility to type 2 diabetes mellitus [48]. In our study, these putative confounding effects might have affected results and the possibility of more conclusive inferences.

Single nucleotide polymorphisms of *SLC2A1*, involved in disease progression in our study, are all intron variants (rs12407920, rs841847, rs841853 and rs3729548). As intronic variants, they cannot possibly cause changes in the protein sequence and they are not associated to alterations of the *SLC2A1* expression. For these reasons, although significant associations were detected in this study, their functional significance seems questionable and their relevance would need further experimental proof. These polymorphisms may not be causative, but linkage disequilibrium with other loci with an etiologic role in diabetic nephropathy cannot be excluded.

In addition, in our study, the sample size was relatively small. The association of diabetic nephropathy and 4 genetic variants in the GLUT1 gene is remarkable. However, the number of patients and controls is low for the inference of genetic association. This is a common phenomenon in candidate-gene association studies [49]. In general, in order to achieve a power >80% for identifying a modest genetic effect (odds ratio 1.2) of a polymorphism present in 10% of the individuals, a sample size of more than 10,000 subjects would be needed [49]. It is obvious that a single institution will never be able to provide a sufficient number of patients to predict association, if it really exists. Then, future collaborative studies may provide more power to detect significant associations by pooling of data. Finally, future meta-analysis of multiple studies may overcome the deficiency of small power and to provide more conclusive evidence for the implication of *SLC2A1* in complications in diabetes [23]. However, the validity of the present findings should be replicated from other gene-candidate or genome-wide association studies (GWAS) [6,39,46,50,51].

Diabetic nephropathy is a complex disease with multifactorial etiology and it involves epistatic and gene-environment interactions, and therefore, single type of genetic studies, such as gene-candidate association studies, have a reduced likelihood to provide conclusive inferences. In addition to hypothesis-driven studies (i.e., the gene-candidate association studies), hypothesis-free studies such as GWAS [23,52,53], microarrays gene expression analyses [54,55] and whole genome linkage scans [56,57] may assist in providing more conclusive evidence regarding the significance of *SLC2A1* as a marker in diabetes leading to complication. This can be achieved by examining the genomic convergence of these different types of studies [53]. Although GWAS represent a superior strategy for unraveling genetic complexity [52], the findings of gene-candidate association studies may be supportive in replicating existed evidence and in revealing genuine genetic effects that could merit prioritization in future studies. However, GWAS themselves lack replication and therefore, replication of their findings from different investigators and different methodologies (such as gene-candidate association studies) are essential to interpret the mass of associations likely to result from GWAS [23,56,57].

However, since the sample size of the present association study was relatively small, we performed a meta-analysis considering all published studies, which investigated the association between *SLC2A1* variants and diabetic nephropathy. In total, eight *SLC2A1* variants were investigated, out of which only five variants were examined in two studies or more and so considered in meta-analysis. Among these five variants, two were also genotyped in our case-control study (rs841853, rs841847). We recorded a significant association between XbaI polymorphism and diabetic nephropathy in analysis of diseased controls versus cases and healthy controls versus cases, after accounting between study heterogeneity using the random effects model. It is noteworthy to mention that meta-analysis when included, our case-control study changed the pattern of results in comparison with healthy controls versus cases, as a significant association was detected for XbaI polymorphism suggesting that this genetic variant maybe associated with diabetic nephropathy.

To the best of our knowledge, it is the most comprehensive meta-analysis regarding *SLC2A1* variants, since it includes all polymorphisms with available data for meta-analysis and all available comparisons between cases, diseased controls and healthy controls. These three comparisons made the trait discrimination more feasible. An important issue in all genetic studies regarding diabetic nephropathy is the demarcation of genetic loci associated with diabetic nephropathy per se and not with the type of diabetes which caused the renal disease. One additional strength of our meta-analysis is the strict definition of cases, as only subjects with persistent moderately increased albuminuria were considered as cases.

However, our meta-analysis has also some limitations. A common issue in meta-analysis is the publication bias, as only published studies were included in meta-analysis. Furthermore, the search was restricted in studies published in English. We should also interpret with caution the results of the meta-analysis because the number of studies is small and the sample size of each study also small.

## Conclusion

In conclusion, the present study presents the results of an association study, which investigated the relation between 14 tag variants across *SLC2A1* and the risk of type 2 diabetes leading to nephropathy and it also reviews the current epidemiology findings regarding the contribution of *SLC2A1* variants in diabetic nephropathy. The results suggest that *SLC2A1* variants and haplotypes may be involved in the pathogenesis of diabetic nephropathy. However, additional studies and a genetic convergence analysis of different data sources are needed in order to merit prioritization in future studies producing more conclusive claims of the association between *SLC2A1* and genetic susceptibility to diabetic nephropathy.
